# Total Lymphocyte Count as surrogate marker for CD4 Cell Count in HIV-Infected Individuals in Gondar University Hospital, Northwest Ethiopia

**DOI:** 10.1186/1742-6405-9-21

**Published:** 2012-07-15

**Authors:** Yitayih Wondimeneh, Getachew Ferede, Gizachew Yismaw, Dagnachew Muluye

**Affiliations:** 1 Unit of ART laboratory, Gondar University Hospital, P.O. Box 196, Gondar, Ethiopia; 2 Department of Medical Microbiology, School of Biomedical and Laboratory Sciences, College of Medicine and Health Sciences, University of Gondar, P.O. Box 196, Gondar, Ethiopia

**Keywords:** Total lymphocyte count, CD4 counts, HAART, Surrogate marker

## Abstract

**Background:**

The high cost of CD4 count estimation in resource-limited countries is a major challenge in initiating patients on highly active antiretroviral therapy (HAART). Therefore, assessment of inexpensive and simple laboratory diagnostic marker is mandatory to diagnose immuno-suppression.

**Objective:**

To evaluate utility of total lymphocyte count (TLC) as surrogate marker for CD4 count in HIV-infected patients.

**Materials and Methods:**

In this cross sectional study, 400 ART-naive HIV-positive patients enrolled in Gondar University Hospital, from March 2011 to May 2011, were tested for CD4 count & TLC. The cutoffs were determined as: 200 cells/μL for CD4 count and 1200 cells/μL for TLC by using BD FACS count and CELL DYN 1800 Flow Cytometrys respectively. Spearman correlation between TLC and CD4 cell count were assessed. Sensitivity, specificity, positive and negative predictive values for different age a group, TLC ≤1200 was computed for CD4 count ≤200 cells/cu.mm.

**Results:**

Among 400 ART naive HIV infected patients, 278 (69.5%) were females. The mean age of the study participants was 33.7. TLC and CD4 count were positively correlated (r = 0.33, p = 0.001). A TLC of ≤1200 cells/m m3 was found to have a sensitivity (32.86%), specificity (95.33%), PPV (79.7%), and NPV (71.9%) for predicting a CD4 count of <200 cells/mm3.

**Conclusion:**

This study showed that low sensitivity and specificity of TLC as a surrogate measure for CD4 count. Moreover, CD4 cell counts of < 200 cells/mm3 were found in 96 cases (24%) with TLCs of ≤1200 cells/mm3. Thus, 1 in 4 individuals would have been deprived of needed treatment. Therefore, we recommend keep on expansion of access to CD4 counter.

## Introduction

The saddle of HIV in resource-limited countries is wide and a large proportion of HIV patients rely on accessing health care services in rural and underserved areas that do not have the capacity or capability to determine CD4 cell counts. Viral loads and CD4 counts require highly skilled laboratory personnel and costly maintenance of complicated equipment [1]. However, Health providers in resource-constrained settings may not have access to this laboratory measurement or its cost may be prohibitive, resulting in the need for an alternative, surrogate marker. Given the decreasing costs and increased availability of antiretroviral therapy (ART) in the developing world, this is an issue of critical and increasing importance [2]. World Health Organization guidelines advocate the use of TLC as a surrogate marker for CD4 cell count [3]. Moreover a number of previous studies indicate that the total lymphocyte count (TLC) may be useful as a surrogate marker of immune status in certain settings [4]. However, controversy regarding the utility of the TLC remains.

The basis for the WHO's suggestion is that most studies concluded a decline in TLC was strongly correlated with a decline in CD4 count, however there were some discrepancies [5]. On the other hand, there is a report which showed due to low sensitivity and specificity, TLC < 1200 cells/mm3 to envisage absolute CD4 count < 200 cells/mm3 was not optimal for identifying patients requiring HAART [6,7]. This showed that the data regarding this issue is still mixed. Moreover, there is also limited information on the relationship between CD4 cell counts and total lymphocyte count in resource-limited settings. In addition, most of the previous studies in different settings were used small sample sizes in HIV- naïve patients. This study was initiated to ascertain the reliability of total lymphocyte count as a substitute for CD4 cell count using relatively large sample size.

## Methods

This cross sectional study was conducted, March 2011 to May 2011 in Gondar University Hospital HIV clinic. This clinic provides care for patients referred from a wide range of primary healthcare facilities in Gondar Town. Using systematic random sampling technique study subjects were selected. The study sample includes HIV-infected adults, 18 years or older and pre-ART HIV positives patients. Study exclusion criteria were antiretroviral therapy and tuberculosis, endocarditis and acute viral infection suspected patients which could affects WBC.

Blood samples were taken from the subjects and hematological indices, such as white blood cell count, and WBC differential count, were determined by automated blood analyzer (CELL-DYN 1800, Abbott Laboratories Diagnostics Division, USA. The CD4 T lymphocytes count was determined using the Becton Dickinson (BD) FASCount system (Becton, Dickinson). The BD FASCount system used flow cytometry for the quantification of the CD4 T Lymphocytes. TLC is easily obtained from the routine complete blood count (CBC) with differential through multiplication of lymphocyte percentage by white blood cell count.

For correlation between CD4 count and TLC, we defined cutoff values as 200 cells/μL and 1200 cells/μL respectively [8], and compared CD4 count with each parameter separately. Data was analyzed in SPSS 16. The correlation coefficient established correlation and kappa coefficient showed agreement between CD4 count and these parameters. Sensitivity, specificity and positive and negative predictive values for using direction on TLC changes as a marker for direction of CD4 changes were calculated. P < 0.05 was considered as statistically significant for all tests.

This study was approved by the ethical committee of Department of Medical Microbiology, Immunology and Parasitology, College of Medicine and Health Sciences, University of Gondar Oral and verbal informed consent was obtained from the patients prior to enrolment. After obtaining consent, demographic questionnaires were completed and blood was drawn for CD4 cell count and CBC.

## Results

Total of 400 ART naive HIV infected subjects were included in this study, among which 278 (69.5%) were females. The mean (standard deviation) age was 33.7 (9.2) years (ranging from 18–70 years). The mean and SD of CD4 count and TLC are shown in Table 1. Comparing with both sexes CD4 count of female patients was higher than male patients. However, there existed no statistical difference in both sexes. Moreover no difference was found between both sexes concerning age and TLC (Table 2). There was positive correlation between CD4 and TLC (r = 0.333, P = 0.001). Among 292 patients, 245 cases had TLC >1200 cells/μL and CD4 > 200 cells/μL, while 47 patients had TLC ≤ 1200 cells/μL and CD4 ≤ 200 cells/μL. In lingering 108 patients, there were no positive correlations between TLC and CD4 count, of whom 96 patients had TLC > 1200 cells/μL, but CD4 < 200 cells/μL and 12 Patients had TLC < 1200 cells/μL, but CD4 > 200 cells/μL. Sensitivity, specificity, PPV, and NPV for TLC cutoff values as compared to CD4 count of <200 cells/mm3 are listed in Table 3. A TLC of ≤1200 cells/mm3 was found to have sensitivity (32.86%), specificity (95.33%), PPV (79.7%), and NPV (71.9%) for predicting a CD4 count of <200 cells/mm3. Kappa coefficient for agreement between CD4 count and TLC was 0.24 fair agreement was observed between CD4 count and TLC (Table 4).

**Table 1:** Mean and range of CD4 count and TLC among HIV infected patients at Gondar University Hospital, 2011

**Marker**	**Mean**	**Median**	**SE Mean**	**SD**	**Range**
CD4 count cells/μL	288	252.5	9.512	190.24	6-1193
TLC cells/μL	2120	1850	57.42	1148.47	350-8480

Abbreviation: CD4, T-lymphocyte CD4 positive; TLC, Total lymphocyte count; SD, standard deviation; HIV, Human immunodeficiency virus.

**Table 2:** Mean of CD4 count and TLC between both sexes among HIV infected patients at Gondar University Hospital, 2011

**Sex**	**Number of patients**	**CD4 (cells/mm**^**3**^**)**	**P -value**	**TLC (cells/mm**^**3**^**)**	**P - value**
male	122	249.26	P=0.45	1982.27	P=0.60
female	278	306.15		2183.60	

**Table 3:** Validity and predictive value between CD4 count and TLC among HIV infected patients in different age categories at Gondar University Hospital, 2011

**Age**	**Marker**	**N**	**P**	**SE**	**SP**	**PPV**	**NPV**	**Kappa**	**P - value**
In all age group	CD4 count ≤ 200 cell/μL	143	35.8	100	100	100	100	0.24	0.001
	TLC ≤ 1200 cell/μL	59	14.8	32.86	95.33	79.66	71.85		
18-29	CD4 count ≤ 200 cell/μL	36	9.0	100	100	100	100		
	TLC ≤ 1200 cell/μL	12	3.0	22.22	95.65	66.66	75.86	0.22	0.002
30-39	CD4 count ≤ 200 cell/μL	66	16.5	100	100	100	100		
	TLC ≤ 1200 cell/μL	33	8.2	39.39	92.92	78.78	69.69	0.35	0.000
40-49	CD4 count ≤ 200 cell/μL	26	6.5	100	100	100	100		
	TLC ≤ 1200 cell/μL	12	3.0	26.92	90.74	58.33	72.05	0.20	0.038
50 & above	CD4 count ≤ 200 cell/μL	16	4.0	100	100	100	100		
	TLC ≤ 1200 cell/μL	7	1.8	43.75	100	100	55	0.38	0.011

Abbreviation: N, number; P, Prevalence (%); SE, Sensitivity (%); SP, Specificity (%); PPV, Positive predictive value; NPV, Negative predictive value; CD4, T-lymphocyte CD4 positive; TLC, Total lymphocyte count; HIV, Human Immunodeficiency virus.

**Table 4:** Agreement between CD4 count and TLC among HIV infected patients at Gondar University Hospital, 2011

**Parameters**		**CD4 count(cell/μL)**		**Kappa**	**Approx. Sig**
		≤200	>200	0.24	0.001
**TLC**	>1200 cell/μL	47	12		
	≤1200 cell/μL	96	245		

Abbreviation: CD4, T-lymphocyte CD4 positive; TLC, Total lymphocyte count; Kappa coefficient for agreement; value < 0.05, significant.

## Discussion

CD4 cell count of ≤ 200 cell/*μ* L is vital marker in the management of HIV/AIDS patients; it is at this stage that antiretroviral therapy (ART) is started and co-trimoxazole prophylaxis is required [9]. Although CD4 cell count is considered the best laboratory marker of HIV infection, it is an expensive test and not widely available because of lack of sophisticated equipment. This problem is more in resource-constrained developing countries where the majority of people infected with HIV are living. To overcome this problem, WHO has recommended that irrespective of the CD4 cell count, ART can be started on patients who have WHO stage III or IV disease and on patients who have WHO stage II disease with TLC of ≤1200/*μ* L (which can substitute CD4 cell count of ≤200/*μ* L), especially in resource-constrained areas [10].

Results of this study demonstrated that there is a positive correlation between CD4 count and TLC. This was in agreement with study conducted by Seyed *et al.*[11]. The present study showed that in three fourths of patients, TLC is a suitable predictor of CD4 count. This finding is consistent with other reports [12,13]. In this study, we found that 24% of patients had TLC > 1200 cells/μL in spite of CD4 < 200 cells/μL that is lower than 38% but higher than 18% reported in Nigeria [14] and Iran [11] respectively*.*

Several studies revealed reasonably adequate sensitivity and specificity to consider TLC as a surrogate measure for CD4 [5,6]. However, this study Supported by the notions of Deresse and Eskindir [15], as we observed low sensitivity and specificity of TLC as an alternate marker to initiate ART. In our study, the sensitivity and specificity of TLC < 1200 to predict CD4 count < 200 were 32.86% and 95.33%, respectively and these figures were lower than what is reported recently from India, 59% and 94%, respectively [7]. As it was reported by Jacobson and colleagues [6], TLC may still be used in resource limited area with the understanding of its low sensitivity and specificity. Stebbing and colleagues also indicated that despite minimally less reliability of TLC as a surrogate for CD4, TLC is important tool in the absence of expensive equipment to measure CD4 [16]. In conclusion, this study showed that low sensitivity and specificity of TLC as a surrogate measure for CD4 count. Moreover, CD4 cell counts of < 200 cells/mm3 were found in 96 cases (24%) with TLCs of ≤1200 cells/mm3. Thus, 1 in 4 individuals would have been deprived of needed treatment. Therefore, we recommend keep on expansion of access to CD4 counter.

## Competing interests

The authors declared no conflicts of interest with respect to the authorship and/or publication of this article.

## Authors’ contributions

YW: Participated in conception and design of the study, data collection, analysis and interpretations of the findings, reviewed the manuscript. GF: Participated in conception and design of the study, analysis and interpretations of the findings, drafting the manuscript and write up. GY: Supervision of the study, analysis and interpretations of the findings, reviewed the manuscript. DM: Participated in analysis and interpretations of the findings, reviewed the manuscript. All authors reviewed and approved the final manuscript.
