# Correction: Total Lymphocyte Count as surrogate marker for CD4 Cell Count in HIV-Infected Individuals in Gondar University Hospital, Northwest Ethiopia

**DOI:** 10.1186/1742-6405-10-2

**Published:** 2013-01-04

**Authors:** Yitayih Wondimeneh, Getachew Ferede, Gizachew Yismaw, Dagnachew Muluye, Meseret Alem, Fanaye Asfaw

**Affiliations:** 1 School of Biomedical and Laboratory Sciences, College of Medicine and Health Sciences, University of Gondar, P.O. Box 196, Gondar, Ethiopia

## Correction

After publication of this work [1], we noted that we inadvertently failed to include the complete list of all coauthors. The full list of authors has now been added and the Authors’ contributions and competing interests section modified accordingly.

## Competing interests

Mr. Meseret and Ms. Fanaye have held supervisions, consultancies and follow up while we are conducting our research. The other authors declare no conflicts of interest.

## Authors’ contributions

YW: Participated in conception and design of the study, data collection, analysis and interpretations of the findings, reviewed the manuscript; GF: Participated in conception and design of the study, analysis and interpretations of the findings, drafting the manuscript and write up; GY: Supervision of the study, analysis and interpretations of the findings, reviewed the manuscript; DM: Participated in analysis and interpretations of the findings, reviewed the manuscript; MA: supervision of the study, consultation, follow up and reviewed the manuscript; FA: supervision of the study, consultation, follow up and reviewed the manuscript

## References

[B1] Yitayih W Getachew F Gizachew Y Dagnachew M Total lymphocyte count as surrogate marker for CD4 cell count in HIV-infected individuals in Gondar University Hospital, North West Ethiopia AIDS Res Ther 2012 9 21 10.1186/1742-6405-9-21 22793790 PMC3522536

